# Prevalence and determinants of adolescent childbearing: comparative analysis of 2017–18 and 2014 Bangladesh Demographic Health Survey

**DOI:** 10.3389/fpubh.2023.1088465

**Published:** 2023-06-19

**Authors:** Nazmul Alam, Mohammad Manir Hossain Mollah, Sharin Shahjahan Naomi

**Affiliations:** ^1^Department of Public Health, Asian University for Women, Chittagong, Bangladesh; ^2^Department of Gender Studies, Asian University for Women, Chittagong, Bangladesh

**Keywords:** adolescent childbearing, geographic inequality, determinants, Demographic Health Survey, Bangladesh

## Abstract

**Objectives:**

Bangladesh has one of the highest adolescent childbearing rates in South Asia, which prevent women from realizing their full potential in life. This study aimed to compare the prevalence and determinants of adolescent childbearing in Bangladesh using data from the 2014 and 2017–18 Bangladesh Demographic and Health Survey (BDHS).

**Methods:**

Nationally representative surveys of respondents were selected using a two-stage sampling process. The study recruited 2,023 and 1,951 ever-married women aged 15–19 from 2014 and 2017–18 BDHS surveys, respectively, from rural and urban settings from all eight geographic divisions of Bangladesh. Univariate and multivariate logistic regression models were fit to determine the factors associated with adolescent childbearing.

**Result:**

The adolescent childbearing prevalence rate was 30.8% in 2014 BDHS and 27.6% in 2017–18 BDHS. Marriage at age 13 years or less also reduced significantly in 2017–18 compared to 2014 (12.7% vs. 17.4%, respectively). Significantly higher odds of adolescent childbearing were found in 2014 among women in the Sylhet Division (adjusted odds ratio (AOR) = 3.0; 95% confidence interval (CI): 1.6–6.1) and the Chittagong Division (AOR = 1.8; 95% CI: 1.8–2.7) compared to the Barisal Region; however, in 2017, there were no significant differences was found across the geographic Divisions. Compared to women in the lowest wealth quintile, women in all other quintiles had lower odds of adolescent childbearing, with the lowest odds found among women in the wealthiest quintile (AOR = 0.3; 95% CI: 0.2–0.6). Women who married at age 14–17 had 60% lower odds of adolescent childbearing compared to the women who married at age 10–13.

**Conclusion:**

Nearly one-third of married adolescents in Bangladesh were pregnant or had at least one child in 2014, and it was reduced only marginally in 2017–18. Marriage at an early age and income inequalities among families were significant predictors of adolescent childbearing in Bangladesh. This study highlighted change in the magnitude and determinants of adolescent childbearing in Bangladesh taken data from two nationally representative surveys conducted 4 years apart.

## Introduction

Remarkable progress has been made globally in reducing early marriage, adolescent childbearing, and maternal mortality over the past few decades, although adolescent childbearing remains a social and public health concern (1–3). The fact that every year approximately 12 million girls age 15–19 give birth in low and middle-income countries is evidence of this grave concern (4, 5). Adolescent pregnancy and childbearing have deleterious consequences at the individual, societal and global levels (6, 7). Adolescent motherhood negatively affects a girl’s health, wellbeing, and educational progress, and prevents her from realizing her full potential (8–12).

Adolescent childbearing is the consequence of complex relationships with multiple factors. High pregnancy rates among adolescent girls are associated with lack of education, less decision-making power in household and family planning, less reproductive knowledge, poverty, and the experience of early marriage (6, 8, 13, 14). The age difference between spouses is also an influential factor in determining early pregnancy (15, 16). Adolescent childbearing rates often are higher in countries where early marriage is prevalent (17, 18). Girls who marry at young ages are more likely to experience multiple pregnancies, recurrent miscarriage, termination of pregnancy, and delivery complications (19, 20). One-third of married teenage girls in Bangladesh become mothers or are pregnant by their 18th birthday (18).

Bangladesh has one of the highest rates of adolescent fertility in South Asia, where 1 girl in 10 has a child before the age of 15 whereas 1 in 3 adolescent becomes mother or pregnant by the age of 19., Adolescent fertility has been one of the widely discussed issues where scientific literature highlighting the social, economic, and reproductive health consequences, particularly connected to a growing risk of intrauterine growth restriction, child undernutrition, preterm birth, and infant mortality (20–23). However, only limited progress has been made thus far mainly because of insufficient attention from policymakers and lack of coordinated efforts by government, non-government, and community-based organizations (24–26). The Government of Bangladesh ratified strict laws against early marriage, however, its success will be grounded on holistic approaches that promote social mobilization and other structural interventions including poverty alleviation, gender equity, and girl’s education that prevent early marriage and reduce the high rate of adolescent pregnancy in Bangladesh (20, 27).

**Figure 1:**
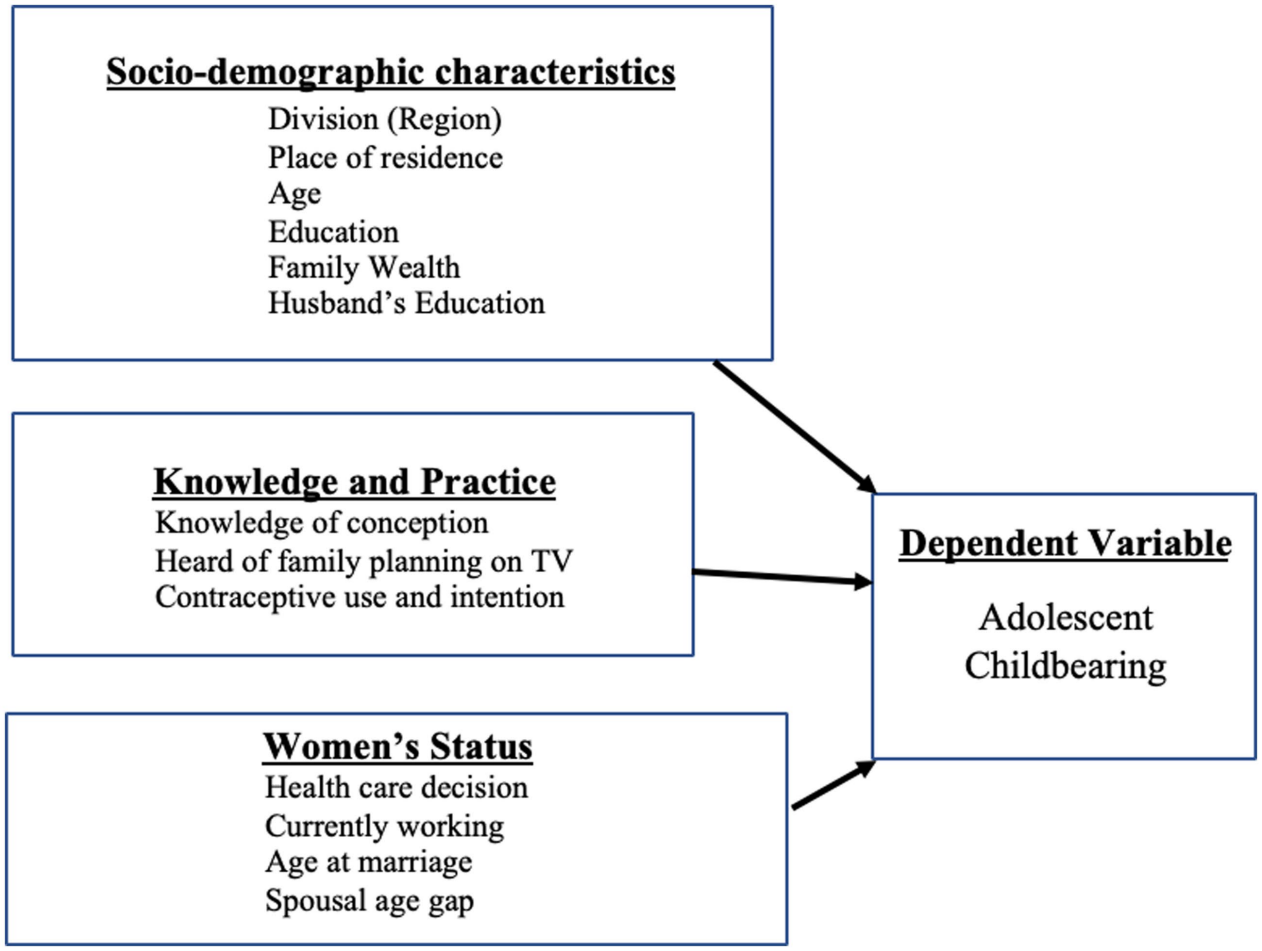
Conceptual framework depicting the linkages among the independent, intermediate, and dependent variables.

Achieving further improvement in lowering adolescent pregnancy rates is a priority for Bangladesh in achieving the Sustainable Development Goals (SDG) targets such as Goal 3 on good health and wellbeing, and Goal 5 on gender equality of girls and women (28). This study compared the prevalence of adolescent childbearing and the factors associated with adolescent pregnancy by using data collected by the nationally representative Bangladesh Demographic Health Survey (BDHS) conducted in 2014 and 2017–18. Independent variables influence the intermediate variables involved with adolescent childbearing in a society. The conceptual framework aids in understanding the role of modifiable and non-modifiable variables on adolescent childbearing (Figure 1).

The study findings will provide evidence of change that has been observed in the prevalence and determinants of adolescent childbearing in Bangladesh between 2014 and 2018. This information will be useful to guide the intervention and policy reform needed to increase the age at first birth and reduce adolescent childbearing in Bangladesh.

## Materials and methods

### Data

This study uses data from the 2014 and 2017–18 Bangladesh Demographic Health Survey, which were the seventh and eighth DHS surveys conducted in Bangladesh. In general, DHS surveys provide information on childbearing, contraception and family planning methods, maternal and child health, and community-level data on the accessibility and availability of health services. All ever-married women age 15–49 in the selected households were eligible to be interviewed. The surveys were designed to produce representative results for the country, urban and rural areas, and for each of the eight administrative divisions. Details about the survey methodology are available in the DHS final report (29). The 2014 BDHS interviewed 17,863 women of age 15–49 and the 2017–18 BDHS survey recruited 20,127 women. Our study samples included 2,023 and 1,951 ever-married women of age 15–19 recruited respectably in 2014 and 2017–18 surveys. BDHS surveys were approved by ICF Institutional Review Board (IRB) and additionally by Bangladesh Medical Research Council (BMRC).

### Variables

The dependent variable was adolescent childbearing, defined as women age 15–19 who were pregnant or had at least one child at the time of interview. Independent variables were: (a) sociodemographic characteristics of the woman and husband; (b) knowledge and practice; and (c) women’s status. Sociodemographic characteristics included division (region), place of residence (urban/rural), family wealth quintile, and woman’s and husband’s education level (no education, incomplete primary, complete primary, incomplete secondary, complete secondary, and higher). Knowledge and practice variables included knowledge of conception, heard about family planning on television in the past few months, and contraceptive use and intention (uses modern method, traditional method, non-user but intends to use, and does not intend to use). Knowledge of conception was recoded as correct knowledge if the respondent answered that the fertile period is in the middle of the women’s cycle and incorrect knowledge for any other answer. Variables that described the women’s status included health care decisions on women’s health care (woman alone, woman and her husband jointly, the husband alone, someone else, and other), working status (currently working or not), age at marriage, and spousal age gap. Age at Marriage was recoded as 10–13, 14–17, and 18–19 years; Knowledge of conception was recoded as no/wrong knowledge, and correct knowledge; spousal age gap was recoded as 2 years or less, 3–5 years, 6–10 years and more than 10 years.

### Statistical analysis

Descriptive analysis was used to assess the sociodemographic characteristics of the study population for the ever-married sample in the surveys. The prevalence of adolescent childbearing was estimated for all ever-married women of age 19 or below by using all women factors. Univariate and multivariate logistic regression models were fit to determine the factors associated with adolescent childbearing. The dependent variable was adolescent childbearing, categorized as a binary variable defined as women age 15–19 who were pregnant or had at least one child at the time of interview. The multivariate logistic models were fit with adjustment of all the independent variables except for the husband’s education, which was found to be highly correlated with the women’s education. -values and 95% confidence intervals are reported to indicate the statistical significance of the odds ratios (OR). Statistical significance was set at *p* value <0.05.

The analysis considered the multi-stage sampling design of the survey and sampling weights. The “all-women factor” was used to adjust the sample weights in this ever-married women sample to make the estimates representative for all women for all analyses except Table 1, which describes the variables among ever-married women. All-women factors are used for adjustments of sampling weight for each woman, multiplying the weight variable for the woman by her appropriate all-women factor to inflate the number of cases from the number of ever-married women to the number of all women.

**Table 1:** Socioeconomic characteristics of adolescent women data from BDHS 2014 and BDHS 2017–18.

Variables	BDHS 2014		BDHS 2017–18		Chi-squared value (value of *p*)
	% *n* = 2023		% *n* = 1951		
Division					
Barisal	6.9	279	5.9	222	
Chittagong	19.8	356	19.3	307	
Dhaka	34.1	339	25.2	293	
Khulna	9.6	276	11.8	259	13.58 (0.0346)
Rajshahi	11.2	270	14	253	
Rangpur	12.1	295	11.6	237	
Sylhet	6.3	208	3.9	148	
Mymensingh	-	-	8.3	232	
Place of residence					
Urban	26.4	629	24.2	612	0.035 (0.85)
Rural	73.6	1,394	75.8	1,339	
Education					
No education	5.1	105	2.2	45	
Incomplete primary	14.9	281	12.6	246	
Complete primary	11.8	236	9.6	188	
Incomplete secondary	50.7	1,037	55	1,060	
Complete secondary	8.3	171	7.1	131	52.36 (0.0001)
Higher	9.2	193	13.5	281	
Family wealth*					
Lowest	19.8	391	19.7	413	
Second	19.7	415	21.2	437	
Middle	21.7	457	22.3	409	6.33 (0.176)
Fouth	22.6	458	21.8	403	
Highest	16.1	302	15	289	
Husband’s education			N = 1886		
No education	14	282	7.6	149	
Incomplete primary	17.4	354	20.6	378	59.14 (<0.0001)
Complete primary	16	315	14.8	294	
Incomplete secondary	32.5	649	32.5	599	
Complete secondary	9.1	176	6.7	132	
Higher	10.9	247	17.4	334	
Knowledge and practice					
Knowledge of conception					
No/incorrect knowledge	81.1	1,640	75.2	1,467	20.10 (<0.0001)
Correct Knowledge	18.9	383	24.8	484	
Heard family planning on TV (*N* = 2021)					
No	81.4	1,642	86.8	1,703	27.26 (<0.00011)
Yes	18.6	379	13.2	248	
Contraceptive use and intention					
Using modern method	45.7	932	42.6	862	
Using a traditional method	4.3	85	5.0	93	
Nonuser-intends to use later	42.5	876	48.4	917	
Does not intend to use	7.4	129	4.0	79	14.78 (0.0020)
Women’s status					
Health care decision (*N* = 1981)			*N* = 1891		
Respondent alone	8.5	147	5.4	100	
Respondent and husband/partner	40.3	760	51.7	979	
Husband/partner alone	35.3	748	26.4	499	110.69 (<0.0001)
Someone else	15.4	315	14.3	266	
Other	0.5	11	2.3	47	
Currently working					
No	83.8	1,731	75.9	1,465	69.23 (<0.0001)
Yes	16.2	292	24.1	486	
Age at marriage					
<=13 Year	17.4	316	12.7	238	
14–17 Year	73.1	1,518	77.3	1,503	10.86 (0.0043)
>17 Year	9.6	189	10	210	
Spouse age gap (*N* = 1978)			N = 1891		
<=2 Years	5.8	128	5.8	114	
3–5 Years	18.5	370	20.3	391	
6–10 Years	46.3	930	47.4	887	2.93 (0.4380)
>10 Years	29.4	550	26.5	499	
Total	100	2,023	100	1951	

* For wealth data, DHS collected detailed information on dwelling and household characteristics and access to a variety of consumer goods, services, and assets, which together gives score to each respondent and then dividing the ranking into five equal parts, from quintile one (lowest-poorest) to quintile five (highest-wealthiest), each having approximately 20% of the population.

## Results

Among ever-married women age 15–19 years, the majority lived in rural areas in 2014 (74%) and also in 2017 (76%). About half of the study subjects had incomplete secondary educations in 2014 (50%) and 2017 (55%), while 5% of the women had no formal education in 2014 reduced to 2% in 2017. Only 16% of the adolescent women were working at the time of interview in 2014 increased to 24% in 2017. Around three-quarters of the adolescent women (81% in 2014 and 75% in 2017) had no or incorrect knowledge of conception. Close to half of the respondents (46% in 2014 and 43% in 2017) reported using modern contraception methods to avoid pregnancy. Health care decisions were made jointly by 40% with husband in 2014 increased to 52% in 2017. Approximately 17% of adolescent women married at age 10–13 in 2014 and reduced to 13% in 2017, while most of them got married at age 14–17 found in both the surveys (Table 1).

In 2014 BDHS, prevalence of adolescent childbearing was found to be 30.8% among women; 29.6% in urban areas, and 31.2% in rural areas. Whereas, in 2017–18, the adolescent childbearing was found to be 27.6%; 27.7% in urban areas, and 27.5% in rural areas. Although it has decreased in 2017–18 compared to 2014, adolescent childbearing remained consistently higher in Rajshahi and Rangpur divisions (37% in 2014 and 33% in 2017–18) compared with other divisions (Table 2).

**Table 2:** Estimates of adolescent childbearing with 95% confidence interval from BDHS 2014 and BDHS 1017–18.

Variables	BDHS 2014		BDHS 2017–18	
	%	95% CI*	%	95% CI*
Division				
Barisal	31.4	28.2–34.6	29.8	25.8–33.9
Chittagong	26.4	23.8–29.1	26.7	23.2–30.3
Dhaka	31.8	27.8–35.7	25.9	22.5–29.3
Khulna	31.2	27.5–35.0	30.4	25.3–35.6
Rajshahi	36.6	32.7–40.5	32.7	27.2–38.1
Rangpur	36.9	32.1–41.7	32.0	28.2–35.8
Sylhet	24.5	21.4–27.5	14.1	10.7–17.4
Mymensingh			30.6	26.4–34.9
Place of residence				
Urban	29.5	26.1–33.0	27.7	24.9–30.6
Rural	31.2	29.3–33.2	27.5	25.7–29.4
Education				
No education	34.9	28.7–41.0	27.6	18.2–37.0
Incomplete primary	33.5	29.5–37.5	32.1	27.1–37.0
Complete primary	31.0	26.4–35.6	33.0	27.9–38.1
Incomplete secondary	30.1	27.7–32.5	25.2	23.2–27.3
Complete secondary	31.3	26.0–36.5	31.6	25.8–37.3
Higher secondary and above	25.9	20.7–31.2	28.2	23.8–32.5
Family wealth				
Poorest	35.1	31.2–38.9	29.0	25.2–32.8
Poorer	32.2	28.8–35.6	28.0	24.8–31.3
Middle	30.0	26.6–33.5	28.7	25.4–32.0
Richer	29.0	26.1–31.9	26.5	23.4–29.5
Richest	27.1	22.7–31.5	24.6	20.8–28.5
Husband’s education				
No education	34.1	29.6–38.6	29.5	22.7–36.4
Incomplete primary	37.7	34.0–41.3	29.0	25.5–32.5
Complete primary	34.1	30.2–38.0	26.6	23.0–30.1
Incomplete secondary	27.3	24.5–30.1	29.8	26.6–33.1
Complete secondary	27.0	21.9–32.1	28.2	22.6–33.7
Higher secondary and above	23.9	20.0–27.8	22.0	18.3–25.7
Knowledge of conception				
No correct knowledge	30.3	28.3–32.3	25.6	23.9–27.2
Correct knowledge	33.3	29.7–37.0	34.8	30.7–38.9
Heard family planning on TV				
No	30.9	29.0–32.9	27.4	25.7–29.1
Yes	30.4	26.9–34.0	28.9	24.8–33.0
Contraceptive method use and intention				
Modern method	34.6	32.2–36.9	31.0	28.1–33.9
Traditional method	21.2	14.5–27.9	22.0	14.9–29.2
Not using but intent to use later	29.5	27.1–32.0	25.7	23.8–27.6
Do not intend to use	22.8	16.3–29.2	22.6	14.9–30.4
Health care decision				
Respondent alone	32.1	25.3–38.8	29.7	21.9–37.4
Jointly	34.7	32.3–37.1	31.3	28.8–33.8
Husband/partner alone	31.1	28.6–33.6	24.0	21.3–26.8
Someone else	22.9	19.2–26.7	23.7	20.3–27.1
Other	11.6	3.9–27.0	21.3	12.5–30.2
Currently working				
No	30.7	28.9–32.5	26.0	24.3–27.7
Yes	31.5	25.7–37.2	33.4	29.5–37.2
Age at marriage				
<=13 Years	32.1	28.5–35.6	26.8	22.6–31.0
14–17 Years	30.5	28.3–32.7	28.0	26.2–29.8
>17 Years	29.8	24.0–35.6	24.4	19.8–29.0
Spouse age gap				
<=2 Years	34.4	28.1–40.7	30.8	23.8–37.7
3–5 Years	30.5	26.7–34.4	25.2	22.1–28.4
6–10 Years	29.6	27.1–32.1	28.2	25.8–30.6
>10 Years	33.3	30.0–36.7	28.2	25.1–31.2

* CI, confidence interval.

Adolescent childbearing decreased consistently with increasing family wealth as found in both survey years. In the 2014 survey, the adolescent childbearing ratio was found to be highest among women with no education (35%) but in 2017–18 it was found highest among women who completed primary education (33%). Similarly, the lowest adolescent childbearing was observed both in 2014 and 2017–18 surveys (26 and 28% respectively) among women whose husbands had higher secondary and above education. Interestingly, adolescent childbearing was found to be lowest among women who reported using traditional methods of contraception in both of the survey years (21 and 22%, respectively). The childbearing was found to be consistently lowest in both survey years when women got married at 18 years and later (30 and 24% respectively). Interestingly, working women had a higher childbearing reported both in 2014 and 2017–18 surveys (32 and 33% respectively) compared to women who did not work.

In the adjusted logistic models from data in the 2014 survey, the highest odds of adolescent childbearing were found in the Sylhet Division (AOR = 3.0; 95% CI: 1.6–6.1) and the Chittagong Division (AOR = 1.8; 95% CI: 1.8–2.7) when compared to the Barisal Division (Table 3). However, in 2017–18 BDHS geographic divisions were not found to be a significant determinant of adolescent childbearing, even though the Chittagong division had the highest adjusted odds (AOR = 1.5; 95% CI: 0.9–2.4). Compared to women in the lowest quintile, women in all other quintiles had significantly lower odds of adolescent childbearing, with the lowest odds found among women in highest quintiles (AOR = 0.3; 95% CI: 0.2–0.6) in 2014 BDHS and (AOR = 0.7; 95% CI: 0.4–1.3) in 2017–18 BDHS, respectively. Women who married at age 14–17 had 60% lower odds of adolescent childbearing and those who married at age 17–19 had 80% lower odds of adolescent childbearing compared to women who married at age 10–13 as found in both 2014 and 2017–18 BDHS. These findings were statistically significant. Women who used traditional contraceptives had 70% lower odds of adolescent childbearing as found in both of the surveys. Women who had 10 plus years of age gap with husband had 1.3 times higher odds having childbearing in 2014 BDHS and 1.5 times higher odds of childbearing in 2017–18 BDHS. However, these findings were not statistically significant.

**Table 3:** Logistic regression of adolescent childbearing of ever-married women age 15–19 years, BDHS 2014 and BDHS 17–18 survey data.

Variables	BDHS 2014		BDHS 2017–18	
	UOR^#^ (95% CI)	AOR^##^ (95% CI)	UOR (95% CI)	AOR (95% CI)
Division				
Barisal	Reference			
Chittagong	1.1 (0.8–1.6)	1.8 (1.2–2.7)**	1.3 (0.8–2.0)	1.5 (0.9–2.4)
Dhaka	1.2 (0.8–1.9)	1.5 (1.0–2.4)**	0.8 (0.5–1.2)	0.8 (0.5–1.3)
Khulna	0.9 (0.6–1.3)	1.1 (0.7–1.6)	0.8 (0.5–1.3)	0.8 (0.5–1.4)
Rajshahi	1.3 (0.9–1.8)	1.2 (0.8–1.8)	0.9 (0.6–1.5)	1.0 (0.6–1.7)
Rangpur	1.4 (0.9–2.2)	1.3 (0.9–2.1)	1.2 (0.7–1.9)	1.1 (0.6–1.8)
Sylhet	2.3 (1.3–4.1)**	3.0 (1.6–6.1)**	1.2 (0.7–2.1)	0.9 (0.5–1.5)
Mymensingh	-	-	1.0 (0.6–1.7)	0.9 (0.5–1.4)
Residence				
Urban	Reference			
Rural	1.3 (0.9–1.8)	0.8 (0.5–1.2)	1.0 (0.7–1.6)	1.0 (0.7–1.5)
Education				
No education	Reference			
Incomplete primary	1.0 (0.5–2.1)	1.2 (0.5–2.8)	1.6 (0.6–4.3)	1.6 (0.5–4.7)
Complete primary	0.8 (0.4–1.8)	1.0 (0.5–2.2)	1.1 (0.4–2.9)	1.2 (0.4–3.6)
Incomplete secondary	0.6 (0.3–1.1)	0.8 (0.4–1.7)	0.6 (0.2–1.5)	0.6 (0.2–1.6)
Complete secondary	0.5 (0.2–1.0)*	0.9 (0.4–1.9)	0.7 (0.3–1.9)	0.8 (0.3–2.4)
Higher	0.3 (0.1–0.5)***	0.6 (0.2–2.3)	0.4 (0.2–1.1)	0.5 (0.2–1.5)
Family wealth				
Lowest	Reference			
Second	0.7 (0.4–1.1)	0.6 (0.4–1.0)	0.7 (0.5–1.1)	0.9 (0.6–1.4)
Middle	0.6 (0.4–1.0)*	0.5 (0.3–0.9)	0.7 (0.5–1.1)	1.0 (0.7–1.6)
Fourth	0.5 (0.4–0.8)**	0.5 (0.3–0.9)	0.6 (0.4–0.9)*	0.8 (0.5–1.4)
Highest	0.4 (0.2–0.6)***	0.3 (0.2–0.6)***	0.5 (0.3–0.8)**	0.7 (0.4–1.3)
Knowledge of conception				
No knowledge	Reference			
Correct knowledge	1.0 (0.7–1.4)	1.2 (0.9–1.8)	1.3 (0.9–1.7)	1.4 (1.0 2.0)
Heard FP on TV				
No	Reference			
Yes	0.8 (0.6 1.1)	1.1 (0.7–1.5)	0.8 (0.6–1.2)	1.0 (0.7–1.4)
Contraception use				
Modern method	Reference			
Traditional methods	0.3 (0.2–0.6)***	0.3 (01.-0.5)	0.4 (0.2–0.7)**	0.3 (0.2–0.6)**
Intend to use later	0.8 (0.6–1.1)	0.9 (0.6–1.3)	0.9 (0.7–1.1)	0.9 (0.7–1.2)
No intention to use	0.6 (0.3–1.0)*	0.5 (0.3–0.9)*	0.4 (0.2–0.8)**	0.3 (0.1–1.0)
Health care decision				
Respondent alone	Reference			
Joint	0.9 (0.5–1.5)	1.0 (0.6–1.6)	1.1 (0.6–2.1)	1.6 (0.9–3.0)
Alone by husband	0.9 (0.5–1.5)	0.9 (0.6–1.5)	0.9 (0.5–1.6)	1.3 (0.7–2.3)
Someone Else	0.**4** (0.2–0.7)**	0.4 (0.2–0.7)	0.8 (0.4–1.4)	1.1 (0.6–2.2)
Currently working				
No	Reference			
Yes	0.7 (0.5–1.2)	0.7 (0.4–1.1)	1.4 (1.1–1.9)*	1.3 (0.9–1.8)
Age at marriage				
<=13 Years	Reference			
14–17 Years	0.4 (0.3–0.6)***	0.4 (0.3–0.7)	0.4 (0.3–0.7)**	0.4 (0.3–0.7)***
>17 Years	0.2 (0.1–0.3)***	0.2 (0.1–0.4)	0.2 (0.1–0.3)***	0.2 (0.1–0.3)***
Spousal age gap				
<=2 Years	Reference			
3–5 Years	0.8 (0.5–1.3)	0.8 (0.5–1.4)	1.1 (0.6–2.0)	1.1 (0.6–2.1)
6–10 Years	0.9 (0.6–1.5)	0.8 (0.5–1.3)	1.3 (0.8–2.2)	1.3 (0.8–2.3)
>10 Years	1.4 (0.8–2.4)	1.3 (0.7–2.2)	1.3 (0.8–2.3)	1.5 (0.8–2.7)

*p ≤ 0.05, **p < 0.01, ***p < 0.001, ^#^Unadjusted odds ratio, ^##^Adjusted odds ratio.

## Discussion

Based on the data of 2014 and 2017–18 BDHS, nearly one-third of married adolescents were either pregnant or already had at least one child before their twentieth birthday, which is substantially higher than in other South Asian countries (7, 9, 22). Considerable progress has been observed in the reduction of adolescent childbearing comparing data from BDHS 2014 and 2017–18, which shows further decline of adolescent childbearing from a study reported BDHS data from 1993 to 2014 (11). Bangladesh remains as one of the leading countries in the world with high rates of teenage childbearing. The high prevalence of adolescent childbearing could be a hindrance for women’s status in Bangladesh because of the negative consequences in health, as well as social and economic wellbeing. Four plausible explanations for high childbearing among adolescents include early age at marriage, early first birth, low contraceptive use among teenagers, and short birth interval (23).

Age at marriage was found to be a significant predictor of adolescent childbearing in this study. Adolescent women who married at age 14–19 had significantly lower odds of adolescent childbearing compared to the women who married at age 10–13. The proportion of adolescent women who got married at age < =13 years reduced substantially comparing data from BDHS 2014 and 2017–18, yet Bangladesh has some of the highest rates of child marriage worldwide, with more than half of all girls being married before the legal age of 18. Girls are often under pressure from both families to give birth to a child early in the marriage (12). Bangladesh enacted Child Marriage Restraint Act in 2017 against early marriage, however, progress has been steady slow over the years because it requires strong complementary social mobilization and other structural interventions that include poverty alleviation, gender equity, and an education program for girls (30). Adolescent women have a lower contraceptive prevalence rate than other women and a higher unmet need for family planning. The prevalence rates of modern contraceptive use rate among adolescent girls age 15–19 was 45.7% in 2014 and 42.6% in 2017–18, compared to 67.7% among women age 25–29 (24, 31). This is important to notice that there has been a little decline of modern methods use among adolescent women between 2014 and 2018.

This study findings suggest that adolescent girls who belong to the highest wealth quintile were significantly less likely to experience adolescent childbearing. Income inequality is a growing source of concern globally contributing negatively to health and developmental outcomes both within and across the countries. The disparity between rich and poor families has influenced overall adolescent health and adolescent childbearing (32). Women from richer households are more likely to have better control over the decision to use contraception and are more aware of the consequences of early childbearing on their own and their children’s health (14, 27). Household wealth could also reflect greater access to media as a tool for better knowledge and awareness including the decision to use contraceptives and reduce adolescent childbearing (13, 33, 34).

There were considerable variations in adolescent childbearing rates across geographic divisions in Bangladesh in 2014 BDHS. After adjusting for the effect of confounding variables, significantly higher odds of adolescent childbearing were found in the Sylhet and Chittagong divisions compared to Barisal Region. These two divisions were reported as low performing regions in Bangladesh for contraceptive use and other maternal and reproductive health indicators (35, 36). The significant variations in adolescent childbearing in these two divisions may be due to differences in religious, cultural, and program coverage (34, 37). However, in 2017–18 BDHS, observed inequality in adolescent childbearing by geographic divisions were not statistically significant. This finding overlays a reflection of an ongoing effort from the government and other stakeholders in reducing regional disparities in adolescent childbearing by introducing regional family planning programs that target the low performing areas in Bangladesh. Adolescents using traditional contraceptive methods have lower fertility than those using modern methods; while working women have higher fertility than those who did not work. These findings are apparently contrary to our understanding; however, sensitivity analyses revealed age of the adolescent was the determining factor in that adolescents who reported using traditional methods were younger in age and thus less likely to experience child bearing and access to modern methods. While currently working adolescents were mostly older and more likely to have a child or being pregnant.

This study has some limitations, both 2014 BDHS and 2017–18 data collected through cross-sectional surveys. So, the causality cannot be ascertained from the predictors as we have discussed in our study findings. However, the DHS data had a large sample size and nationally representative sampling methodology, which increase the generalizability of our study findings. Besides, some unmeasured characteristics including parental socioeconomic status and cultural influences on childbearing after marriage might have a systemic influence on adolescent childbearing in Bangladesh.

## Conclusion

This study has offered a comparison of the magnitude and determinants of adolescent childbearing in Bangladesh in terms of economic, social, and reproductive factors taken data from two nationally representative surveys conducted 4 years apart. This study highlighted that the adolescent girls from the highest wealth quintile had lower odds of childbearing during their adolescent period. Age at marriage was found to be a significant determinant of adolescent childbearing. To address the issues of family wealth inequality, and child marriage, it is paramount to strengthen structural interventions that target individual and societal level change and raise awareness, poverty alleviation, girls education, and employment (38). Bangladesh is one of the few developing countries that has achieved most of the Millennium Development Goals that include reducing poverty, increasing female education, and reducing gender inequality (2, 18, 28). The study findings highlighted that Bangladesh is making progress in reducing adolescent childbearing among married adolescent women. The study findings could be useful in formulating policies and interventions that address modifiable factors to further reduce adolescent childbearing and achieve improved health and psychosocial wellbeing for women in Bangladesh.

## Data availability statement

The datasets presented in this study can be found in online repositories. The names of the repository/repositories and accession number(s) can be found at: https://dhsprogram.com/.

## Ethics statement

Bangladesh Demographic Health Surveys were reviewed and approved by Bangladesh Medical Research Council Ref: BMRC/NREC/2016-2019/324. Written informed consent to participate in this study was provided by the participants themselves or participants’ legal guardian/next to kin.

## Author contributions

NA, MM, and SN conceptualized the study and interpreted the data and prepared the final manuscript. MM and NA analyzed the data. All authors contributed to the article and approved the submitted version.

## Funding

No funding was needed for this study, which used secondary data from Bangladesh Demographic Health Survey (BDHS). The DHS Program (#AID-OAA-C-13-00095) was implemented with support from the United States Agency for International Development (USAID).

## Conflict of interest

The authors declare that the research was conducted in the absence of any commercial or financial relationships that could be construed as a potential conflict of interest.

## Publisher’s note

All claims expressed in this article are solely those of the authors and do not necessarily represent those of their affiliated organizations, or those of the publisher, the editors and the reviewers. Any product that may be evaluated in this article, or claim that may be made by its manufacturer, is not guaranteed or endorsed by the publisher.
