# Supplementary material for: Prevalence and determinants of adolescent childbearing: comparative analysis of 2017–18 and 2014 Bangladesh Demographic Health Survey
Source: Front Public Health. 2023 Jun 19;11:1088465. doi: 10.3389/fpubh.2023.1088465 (PMC10315475; doi:10.3389/fpubh.2023.1088465)
Supplement: Supplementary file 1 [file Table_1.DOCX]

Sensitivity analysis

Table 1: Participants age by their working status

|  | Currently working | |  |
| --- | --- | --- | --- |
| Age in years | Yes  N (%) | No  N (%) | Chi-squared value, p value |
| 15 | 18 (9.5) | 172 (90.5) | 24.98 (0.0001) |
| 16 | 34 (11.7) | 257 (88.3) |  |
| 17 | 50 (11.6) | 381 (88.4) |  |
| 18 | 75 (13.7) | 473 (86.3) |  |
| 19 | 115 (20.4) | 448 (79.6) |  |

Table 2: Participants age by their use of contraceptive methods

|  | Use of Contraception | |  |  |
| --- | --- | --- | --- | --- |
| Age in years | Modern Methods  N (%) | Traditional Methods  N (%) | Nothing  N (%) | Chi-squared value, p value |
| 15 | 84 (44.2) | 9 (4.7) | 97 (51.1) | 26.84 (0.008) |
| 16 | 128 (43.9) | 17 (5.8) | 144 (50.3) |  |
| 17 | 198 (45.9) | 8 (1.9) | 225 (52.2) |  |
| 18 | 236 (43.1) | 29 (5.2) | 283 (51.7) |  |
| 19 | 286 (50.8) | 22 (3.9) | 255 (45.3) |  |

Table 3: Participants age by their fertility status

|  | Fertility | |  |
| --- | --- | --- | --- |
| Age in years | Yes  N (%) | No  N (%) | Chi-squared value, p value |
| 15 | 76 (40.0) | 114 (60.0) | 132 (0.0001) |
| 16 | 154 (52.9) | 137 (47.1) |  |
| 17 | 280 (64.9) | 151 (35.1) |  |
| 18 | 396 (72.3) | 152 (27.7) |  |
| 19 | 445 (79.4) | 118 (20.6) |  |

Table 4: Participants fertility status by their working status.

|  | Fertility | |  |
| --- | --- | --- | --- |
| Currently working | Yes  N (%) | No  N (%) | Chi-squared value, p value |
| Yes | 203 (69.5) | 89 (31.5) | 1.15 (0.283) |
| No | 1148 (66.3 | 583 (33.7) |  |
